# 

**Published:** 1889-10-26

**Authors:** 


**NOTICE TO CORRESPONDENTS.**

All MS., Letters, Books for Review, and other matters intended for the Editor, should be addressed The Editor, The Lodge, Porchester Square, London, W.

**Notice to Advertisers and Subscribers.**

All Advertisements, Orders for Copies of The Hospital, and Business Communications should be addressed to The Publisher, *not to the Editor*, The Hospital, 139-140, Salisbury-court, Fleet-street, E.C.

### BOOKS RECEIVED.

W. H. Allen and Co.: "The Diseases and Disorders of the Ox," by George Cresswell.

Baillière, Tindall, and Cox: "Cancer and its Complications," by C. E. Jennings, F.R.C.S., M.S., M.B.

George Bell and Sons: "Harvey's Circulation of the Blood," by Alex. Bowie, M.D., C.M.

J. and A. Churchill: "The Management of Children," by a Mother. Second edition.

H. K. Lewis: "Hygiene of the Nursery," by Louis Starr, M.D. Second edition, illustrated.

Religious Tract Society: "Ralph Ellison's Opportunity," by Leslie Keith.

Smith, Elder, and Co.: "Artificial Feeding, and Food Disorders of Infants," by W. B. Cheadle, M.A., M.D., F.R.C.P.

Swan Sonnenschein and Co.: "A Manual of Home Nursing," by Louisa Emily Dobree.

Report of the Pasteur Institute, Mansion House Fund; *Brooklyn Medical Journal*, vol. iii., No. 9; Second and Third Annual Reports of the Association for the Advancement of Boarding Out; "Boarding Out," by Welbelmina L. Hall, F.R.Met.Soc. (4d.)

### SCRAPS AND GLEANINGS.

Hospital Sunday at Wisbech brought in £9.

Dr. Hadden has been elected surgeon to Wexford Infirmary.

"Truth" Toy Show will be held at the Grosvenor Gallery on Dec. 16 and 17.

The workmen of Steiner's Mills have sent £134 to the Blackburn Infirmary.

The collection at Ewell Church after the harvest festival service was for the Sick Nurse Fund.

The Suffolk papers are full of letters on the subject of the cloud over the Suffolk General Hospital.

An anonymous lady donor has given £3,000 to the North-East Lancashire Deaf and Dumb Society.

The Medical Officer of Health for Epsom desires the board to secure an infectious diseases hospital.

The foundation-stone of the new buildings of the New York Academy of Medicine was laid on Oct. 2nd.

The cholera epidemic in Persia is decreasing. The mortality at Kermanshah reached a total of 500.

Diphtheria is still carrying off over 30 victims a week in London, whereas scarlet fever only claims 15 victims weekly.

A patient in the Hotel Dieu, Paris, is said to have twofold vision. She can see two different sets of objects at the same time.

A Bengali paper affirms that premises fumigated with dhuna (the resin of the Shorea Robusta plant) are never entered by snakes.

The number of suicides amongst physicians have been extraordinary lately. Dr. Charles Royston, of Bayswater, is one of the latest victims.

Grimsby Hospital issues its annual report. In spite of reduced expenses, the committee have failed to overtake the arrears, which amount to £600.

The Prince of Wales has consented to open the Royal Victoria Hospital, Bournemouth, on January 16th. It is hoped that the Princess will also be present.

The Provident Surgical Appliance Society, of 12, Finsbury-circus, has been awarded a silver medal at Paris Exhibition for artificial limbs and surgical appliances.

Reigate Cottage Hospital issues a satisfactory report, which states that a Samaritan Fund is being formed. Nearly 200 patients were treated during the year.

Mr. John Farrington, M.R.C.S., L.R.C.P., has been appointed house surgeon to the Pendleton Branch of the Salford and Pendleton Royal Hospital and Dispensary.

A Guernsey youth having a birching in prospect, took iodine with the idea of making himself too ill to receive corporal punishment. The freak nearly terminated fatally.

The late Professor Elias Loomis, of Yale, has bequeathed to the university the greater part of his estate, which is valued at from 250,000 to 300,000 dols. (£50,000 to £60,000).

A child, aged four years, lately died after moistening transfer pictures with his tongue. His death was supposed to be due to the introduction of an irritant poison into the system.

Dr. Woodhouse, of Belfast, says 10 per cent. of the death-rate is attributed to diarrhœa in infants, and he wishes more lectures were given to the poor on the proper feeding of children.

The Eastbourne guardians, after an animated debate on vaccination, have decided to commence anti-vaccination prosecutions similar to those which caused some commotion three years ago.

Samuel Coles, whose age, as attested by the present register, was 105 years, died last week at Laxton's Hospital, Northamptonshire. He used to drink two glasses of beer daily, but was not a smoker.

Alderman Bennett, a prominent member of the Manchester Corporation, died in Charing Cross Hospital last week. He had been mayor of the borough and president of the Chamber of Commerce.

The quarterly report of Norfolk and Norwich Hospital makes mention of the amusement provided to patients and nurses by the bands which have played in the hospital gardens during the summer.

A special clause in the Infectious Diseases Act, which comes into operation on the 1st of November, requires that a medical officer of health shall be elected in the parish of Woolwich, as in all other parts of the kingdom.

Dr. Hadlich, a physician belonging to Pankow, a suburb of Berlin, who had been living for some weeks by the Lake of Geneva, started several days ago on an excursion in the Alps. He has not returned, and nothing has since been heard of him.

Mr. C. R. M. Talbot, M.P., "father of the House of Commons," and Lord-Lieutenant of Glamorganshire, has forwarded a cheque for £1,000 to the secretary of the Home of Rest for Invalids and the Distressed Poor, at Porthcawl. Mr. Talbot entered Parliament in 1830.

A case of leprosy has just been reported to the Romford rural sanitary authority. The sufferer is a German seaman named Otto, living at Great Warley, near Brentwood. The greater part of his life has been spent upon the seas in various parts of the globe, and it is supposed he contracted the disease abroad.

The Guild of St. Luke, a guild entirely composed of medical men, founded for evangelistic work among their patients, held its annual festival on Oct. 18th, at St. Paul's Cathedral. The seats beneath the famous dome were fully occupied by a crowd of some two or three thousand persons interested in the work of the guild. Dr. Dover preached.

At last Saturday's meeting of the Metropolitan Asylums Board, it was stated that in view of the fact that 42 patients were admitted on Thursday and 39 on Friday, the South-Western Hospital has been thrown open, and there are 373 patients at the Eastern Hospital, 230 at the North-Western Hospital, 194 at the Western Hospital, 341 at the South-Eastern, and 413 at the Northern Hospital.

